# Health-Promoting Behaviors, Risk Perceptions, and Attention to COVID-19-Related Information: Comparing People's Responses to the COVID-19 Pandemic Across Times of Chinese New Year and Summer 2020 in Hong Kong

**DOI:** 10.3389/fpubh.2021.688300

**Published:** 2021-11-23

**Authors:** Yue Xu, Hui-Fang Chen, Wai Keung Jerf Yeung, Chih-Wei Hsieh, Hsiang-Yu Yuan, Lennon Yao-Chung Chang

**Affiliations:** ^1^Department of Social and Behavioral Sciences, City University of Hong Kong, Kowloon, Hong Kong SAR, China; ^2^Department of Public Policy, City University of Hong Kong, Kowloon, Hong Kong SAR, China; ^3^Department of Biomedical Sciences, City University of Hong Kong, Kowloon, Hong Kong SAR, China; ^4^School of Social Sciences, Monash University, Clayton, VIC, Australia

**Keywords:** health-promoting behaviors, COVID-19, risk perceptions, fear, attention to COVID-19-related information

## Abstract

**Background:** Since the start of the COVID-19 pandemic, individuals have been encouraged to engage in health-promoting behaviors, namely actions taken to prevent infection and keep themselves healthy, such as maintaining social distancing. However, other factors, such as risk perception and feelings of fear, also might influence whether an individual takes such measures. This study compared people's responses to the pandemic in terms of their adoption of COVID-19 health-promoting behaviors, COVID-19 risk perceptions, and attention to COVID-19-related information during two periods: the 2020 Chinese New Year (CNY) in Hong Kong (HK), i.e., the very beginning of the COVID-19 outbreak (Time 1, T1), and summer 2020, i.e., before and during the third wave of COVID-19 infections in HK (Time 2, T2).

**Methods:** Data were extracted from 180 HK participants, who were asked to recall and report their health-promoting behaviors, emotional and cognitive COVID-19 risk perceptions, and attention to COVID-19-related information during T1 and T2. A repeated-measures ANOVA series was conducted to investigate differences in public responses between the two aforementioned time points.

**Main Findings:** After controlling for the effects from gender, age, and education levels, the participants reported practicing more infection-prevention behaviors, experiencing a lower level of fear as a psychological response, and paying less attention to COVID-19-related information during T2 than T1.

**Conclusions:** This study addressed the need to monitor public responses to the COVID-19 pandemic, including changes in people's behaviors and psychological responses across time. The results also suggest that the HK public was steered toward striking a balance between strengthening their infection-prevention behaviors and reducing their fear of COVID-19 infection.

## Introduction

Coronavirus disease 2019 (COVID-19) is a severe respiratory disease, and its virus, severe acute respiratory syndrome coronavirus 2 (SARS-CoV-2; henceforth COVID-19), can be transmitted easily among people (1). The first COVID-19 outbreak was reported in Wuhan in 2019, and the disease eventually spread globally, prompting the World Health Organization (WHO) to declare the COVID-19 outbreak a global pandemic in March 2020 (2, 3). In Hong Kong (HK), the COVID-19 outbreak started toward the end of January 2020, around the period of the Chinese New Year (CNY) Festival. The first two cases were confirmed on January 23, 2020, a day before the CNY Festival (4). An emergency response level, the highest warning tier, was announced on January 25, 2020 (5). In March 2020, the second wave of infections began in HK, with imported cases being the primary sources this time around. HK experienced the third wave of community infections from early July to August 2020 (6).

Health-promoting behaviors refer to self-initiated actions that aim to control and improve health and prevent diseases (7, 8). Considering that COVID-19 is highly contagious, it has posed a grave threat to healthcare systems worldwide (9). To prevent or control the spread of COVID-19, governments have encouraged individuals to take infection prevention and control measures, such as wearing surgical masks and maintaining social distancing. As adopting one behavior alone is insufficient, individuals often have been encouraged to embrace all aforementioned measures to prevent COVID-19 infection (10). For example, both wearing a medical mask and maintaining physical or social distancing can be viewed as the best ways to prevent pathogen exposure (11). Also, adopting some health-promoting behaviors (e.g., adopting a healthy diet and exercising) to keep oneself healthy can help people cope with stress, strengthen their immune systems, and reduce negative impacts on health from some infection-prevention behaviors (e.g., isolation and quarantine) (12–14). Thus, it is important to investigate public adherence to governments' call for infection-prevention behaviors during different COVID-19 outbreak stages.

Previous experiences might influence people's behaviors and attitudes toward new diseases. HK experienced the Severe Acute Respiratory Syndrome (SARS) outbreak in 2003, during which the public adopted similar health-promoting behaviors to prevent infection. Research found that more than 60% of survey respondents in HK consistently wore masks to prevent infection during the 2003 SARS outbreak (15). Hong Kong residents could have associated the COVID-19 pandemic with their SARS experiences, making them more vigilant about taking protective measures against COVID-19. However, the public's adherence to the recommended behaviors could change due to factors such as policy changes and the pandemic's severity. For example, guidelines on wearing a mask as an infection-prevention measure have changed over time (16, 17), which may lead to changes in the public's behavior. Researchers have not yet fully determined whether the public's adherence to recommended behaviors changes over time. Furthermore, HK's government implemented stricter policies as the COVID-19 outbreak became a global pandemic, such as restrictions on gatherings (18, 19). Hong Kong residents might accept more health-promoting behaviors if the pandemic worsens, but the literature has not addressed any such changes yet.

Risk perception is an essential concept in health behavior theories and works as a potential motivator for one's infection-prevention behaviors (20, 21). Risk perception has been investigated in relation to previous disease outbreaks, such as SARS, Ebola, and avian influenza (22–24). Examining the public's risk perceptions will facilitate the investigation of the public's adherence to recommended infection-prevention behaviors, and a thorough understanding of the vital determinants of behavior also will increase the ability to promote infection-prevention behaviors among the public (25). The health-belief model proposes that perceived susceptibility (likelihood of having a disease) is one of the key constructs in predicting individuals' behaviors (26). Although some empirical findings were mixed, most studies highlighted risk perception's role in predicting corresponding behaviors. For example, Brug et al. (27) found that the perceived risk of being infected by SARS was related positively to worries about SARS and resulting infection-prevention behaviors. In other words, a positive relationship exists between cognitive risk perception and health behaviors, implying that the public would show higher perceived vulnerability later during the COVID-19 outbreak than earlier.

Previous research investigated risk perception from cognitive and emotional perspectives (20, 21). The cognitive domain of risk perception pertains to one's perception of likely or potential risk outcomes by processing these behaviors in a deliberate and logical way through reasoning (21). In the present study, we not only assessed HK participants' overall risk perception, but also investigated their perceived outing risk (perceived likelihood of being infected if one did not adopt specific infection-prevention behaviors) and community risk (perceived increased likelihood of infection in one's community) to study the cognitive domain of risk perception. Behavior-specific outing risk is related more closely to particular infection-prevention behaviors than to overall risk perception (20). During the SARS pandemic, a community outbreak infected 321 residents of Amoy Gardens, a large apartment complex in HK (28). Thus, HK residents may perceive the risk of community transmission possibly leading to a severe outbreak. Indeed, during the first COVID-19 outbreak, many communities' residents held rallies in which they expressed their disapproval over the designation of COVID-19 hospitals or quarantine facilities in their own communities (29, 30).

Emotion is another domain of risk perception, referring to one's intuitive reaction to dangerous events (20, 21, 31), and fear is one of the emotional responses to acute threats posed by the COVID-19 pandemic (32, 33), which might amplify risk estimates. Lerner and Keltner (34) used the appraisal tendency approach to indicate emotion-specific influences on judgment and choices. They explained how people experiencing fear tend to perceive negative events as unpredictable and establish control depending on situations. Empirically, risk perception is related closely to fear of a disease outbreak. For example, Yang and Chu (23) found that fear and anxiety are associated positively with Ebola risk perception. Moreover, fear and anxiety can be viewed as motivators for individuals to adopt infection-prevention behaviors, and some studies have found a positive association between fear and infection-prevention behaviors (35, 36). However, a high level of fear also may increase the risk of developing mental health issues, such as psychological distress and insomnia (37), depression and anxiety (38), and even suicidal ideation (39). Because previous research has pointed out the association between fear and taking preventive measures, and its potential impact on mental health, this research will compare the public's responses while experiencing fear at different time points.

Excluding risk perception, the public attention to COVID-19-related information might be an important indicator of taking health actions and experiencing emotional reactions to COVID-19. Considering that individuals might acquire updated knowledge about COVID-19 to learn how to take action against it, it is important to understand public attention to COVID-19-related information. An extant study compared Google searches for “hand washes” and “face mask” in different countries early on during the COVID-19 pandemic and found that the number of “hand washes” searches was associated negatively with the speed of COVID-19 spread (40). However, although some may have obtained knowledge about COVID-19 during the outbreak's initial stages, knowledge about anti-COVID-19 measures has evolved continuously (e.g., infection-prevention behaviors' effectiveness over time), thereby requiring public attention. Bento et al. (41) found an increased number of “COVID-19” searches after the first confirmed case in the U.S., but the number of searches went back to normal in a few days. However, media exposure to COVID-19-related information also may be related to fear and anxiety (42). For example, Husnayain et al. (43) found that monitoring Google searches using COVID-19-relevant keywords could be a way to monitor the state of public restlessness amid the pandemic. Considering that COVID-19 infections have been up and down in HK, and that very few studies have been conducted on COVID-19 that aimed to monitor public attention to COVID-19-related information, the present study intended to address this issue. As the public was exposed to COVID-19 for around half a year between CNY and the summer of 2020, the public may have become desensitized to the disease (44). We hypothesized that HK residents paid less attention to the pandemic over time and experienced less fear as a psychological and physiological response.

The present study aimed to compare changes in public responses to recommended health-promoting behaviors, cognitive and emotional domains of the public's COVID-19 risk perceptions, and public attention to COVID-19-related information in HK during two periods: CNY and summer 2020. As Bish and Michie (45) suggested that gender, age, and education levels might influence people's infection-prevention behaviors, we controlled for the impact from these factors and examined the following: (H1) whether participants would adopt more infection-prevention behaviors and other such behaviors to remain healthy during summer 2020 than during CNY; (H2) whether participants would perceive more likelihood of being infected, outing risk, and community risk during summer 2020 than during CNY; and (H3) whether participants would experience less fear as a psychological and physiological response and pay less attention to COVID-19 during summer 2020 vs. during CNY.

## Materials and Methods

### Study Participants

Altogether, 266 participants completed the survey questionnaire, but only 180 were in HK for both CNY and during the week when they completed the survey during summer 2020. Therefore, only these 180 participants were eligible for the present study, and their data were analyzed and reported. These participants ranged from 18 to 65 years old [mean = 29.0; standard deviation (SD) = 11.9]. Nearly 78% were single, 58% held bachelor's degrees, 76.7% were permanent HK residents, and 50% were males. Furthermore, 9 out of the 180 respondents were under compulsory quarantines, and 28 quarantined voluntarily at home for 2 weeks or more. Demographic data are provided in Table 1.

**Table 1:** Descriptive statistics of the study participants' demographic information (*N* = 180).

	***N***	**Percent**
**Gender**		
Female	89	49.4
Male	90	50
Missing data	1	0.6
**Age**		
18–29	122	67.8
30–41	27	15.0
42–53	14	7.8
54–65	13	7.2
Missing data	4	2.2
**Marital status**		
Single	140	77.8
Married	36	20.0
Divorced	3	1.7
Missing data	1	0.6
**Education level**		
High school	14	7.8
Undergraduate degree	105	58.3
Master's degree	47	26.1
Doctoral degree	14	7.8
**Permanent residency**		
Hong Kong	138	76.7
Mainland China	41	22.8
Others	1	0.6
**Voluntary quarantine at home**		
Yes	28	15.6
No	151	83.9
Missing data	1	0.6
**Compulsory quarantine**		
Yes	9	5.0
No	170	94.4
Missing data	1	0.6

### Procedures

The Institutional Review Board (IRB) and Human Subjects Ethics Sub-Committee of City University of Hong Kong approved the study (Application No. H002392). The survey was administered between June 11 and August 10, 2020. All participants were recruited through convenience and snowball sampling *via* email or in-person invitations. Their participation was voluntary, i.e., not secured through any incentives. They either answered an online survey *via* Qualtrics software (Qualtrics, Provo, UT) or in paper form. All participants provided consent and confirmed their eligibility for participation in the study (i.e., they were in HK during the past week and were older than 18) before the study started. It took around 10–15 min for participants to complete the questionnaire.

The participants rated their responses to recommended health-promoting behaviors, COVID-19 risk perceptions, and attention to COVID-19-related information during CNY 2020 (Time 1, T1) and during the week before they completed the survey (Time 2, T2). At the start of CNY 2020, four pictures about CNY (see Supplementary Material) were shown to the participants as a priming method to help them recall what they did and felt during CNY 2020. The participants also provided their demographic information at the end of the survey.

### Measurements

#### Health-Promoting Behaviors

Items were adapted from previous studies (27, 46) conducted to examine the public's health-promoting behaviors during the SARS outbreak. The present study measured two types of health-promoting behaviors: *infection-prevention behaviors* (eight items) and *keeping healthy* (four items), as provided in the Supplementary Material. *Infection-prevention behaviors* aimed to prevent COVID-19 infection, e.g., wearing masks; avoiding crowded public places, including restaurants; and washing hands frequently. *Keeping healthy* refers to behaviors that maintain one's personal health, e.g., maintaining a healthy diet and exercising, avoiding excessive stress, and having regular and ample sleep. The participants were asked to rate these items on a five-point scale from 1 (not corresponding strongly) to 5 (corresponding strongly). Cronbach's alpha values for infection-prevention behaviors were 0.90 during T1 and 0.83 during T2, with those for keeping healthy at 0.79 during T1 and 0.85 during T2.

#### COVID-19 Risk Perceptions

Altogether, 19 items were used to assess the cognitive and emotional domains of the participants' COVID-19 risk perceptions. The participants were asked to rate their agreement with each of the items on a scale from 1 (“strongly disagree”) to 5 (“strongly agree”). The items in the cognitive domain were developed on the basis of Dillard et al.'s (47) suggestion that the participants indicate the degree of their agreement with each item on a Likert scale, and on Brewer et al.'s (21) suggestion that conditioned risk questions be used to assess risk perceptions. Seven items were adapted from previous studies that examined cognitive risk perceptions during disease outbreaks (47, 48). For the items in the emotional domain, the present study adopted the items developed by Ahorsu et al. (49) to investigate fear as a psychological and physiological response to the COVID-19 pandemic. We also added one item to the scale to measure participants' physiological response: “When I thought that I might have been infected with COVID-19, my appetite became worse.”

As indicated in the Supplementary Material, the cognitive and emotional domains of COVID-19 risk perception were measured in terms of likelihood of being infected, outing risk, community risk, and psychological and physiological responses. The Cronbach's alpha values were 0.88, 0.83, 0.88, 0.82, and 0.92 during T1 and 0.90, 0.88, 0.91, 0.86, and 0.96 during T2, respectively.

#### Attention to COVID-19-Related Information

Two items were adapted (50) to measure the participants' attention to COVID-19-related information. The participants were asked whether they paid attention to COVID-19 news and searched for information about COVID-19 during CNY 2020 and during the week before they completed the survey, and they answered on a scale from 1 (“strongly disagree”) to 5 (“strongly agree”). The Cronbach's alpha was 0.76 during T1 and 0.79 during T2.

### Data Analysis

Data analyses were conducted using SPSS 24.0 for Macintosh (51). The average scores for each factor were calculated.

#### Hypotheses Testing

Repeated measures ANOVAs were conducted in each pair among the participants in HK during both T1 and T2 (*N* = 180) to determine whether significant differences existed in the aforementioned variables between T1 and T2. The sociodemographic variables (e.g., age, gender, and education level) were treated as control variables in the analyses. Education level was recoded as follows: high school and undergraduate levels were combined into one group, and master's and doctoral degree levels were combined into another group.

## Results

Table 2 provides information on health-promoting behaviors, risk perception, and attention to COVID-19-related information at two time points after controlling for age, gender and education level. The participants practiced more infection-prevention behaviors [*F*_(1, 169)_ = 9.66, *p* = 0.002, $\eta_{\text{p}}^{2}$ = 0.05] during summer 2020 than during CNY, but did not report greater efforts to remain healthy [*F*_(1, 170)_ = 2.87, *p* = 0.092, $\eta_{\text{p}}^{2}$ = 0.02].

**Table 2:** Results of repeated measure ANOVAs of the differences in health-promoting behaviors, COVID-19 risk perceptions, and attention to COVID-19-related information.

		**T1**	**T2**		
	***N***	**Mean (SD)**	**Mean (SD)**	***F***	** ${\text{η}}_{\text{p}}^{2}$ **
**Health-promoting behaviors**					
Infection prevention behaviors	174	4.16 (0.79)	4.27 (0.60)	9.66^**^	0.05
Keeping healthy	175	3.53 (0.84)	3.75 (0.85)	2.87	0.02
**COVID-19 risk perception**					
Likelihood of being infected	175	4.23 (0.66)	4.26 (0.67)	2.27	0.01
Outing risk	175	3.61 (0.77)	3.74 (0.83)	0.16	0.00
Community risk	175	4.13 (0.82)	4.13 (0.85)	0.84	0.01
Psychological responses of fear	175	3.25 (0.93)	2.86 (1.03)	23.31^***^	0.12
Physiological expressions of fear	175	2.01 (1.00)	1.97 (1.06)	2.45	0.01
**Attention to COVID-19-related information**					
	175	4.08 (0.89)	3.62 (1.01)	22.44^***^	0.12

* *p < 0.05;*

** *p < 0.01;*

*** *p < 0.001*.

Regarding risk perceptions, no significant differences were found in the cognitive domain at two time points, including perceived likelihood of being infected [*F*_(1, 170)_ = 2.27, *p* = 0.133, $\eta_{\text{p}}^{2}$ = 0.01], outing risk [*F*_(1, 170)_ = 0.16, *p* = 0.686, $\eta_{\text{p}}^{2}$ = 0.00], and community risk [*F*_(1, 170)_ = 0.84, *p* = 0.360, $\eta_{\text{p}}^{2}$ = 0.01]. It is notable that participants scored very high on likelihood of being infected (mean = 4.23, SD = 0.66 during T1; mean = 4.26, SD = 0.67) and community risk (mean = 4.13, SD = 0.82 during T1; mean = 4.13, SD = 0.85 during T2) at two time points. Regarding the emotional domain of risk perception, significantly lower psychological responses from fear were shown during T2 compared with T1 [*F*_(1, 170)_ = 23.31, *p* < 0.001, $\eta_{\text{p}}^{2}$ = 0.12]. However, participants reported few physiological expressions of fear (mean = 2.01, SD = 1.00 during T1; mean = 1.97, SD = 1.06 during T2) at two points, with no significant differences over time [*F*_(1, 170)_ = 2.45, *p* = 0.120, $\eta_{\text{p}}^{2}$ = 0.01].

Finally, the participants reported paying significantly less attention to COVID-19-related information during T2 than during T1 [*F*_(1, 170)_ = 22.44, *p* < 0.001, $\eta_{\text{p}}^{2}$ = 0.12].

## Discussion

This study was conducted to compare changes in public adoption of health-promoting behaviors, cognitive and emotional domains of the public's COVID-19 risk perceptions, and public attention to COVID-19-related information during CNY and summer 2020 in Hong Kong. The results indicate that the participants adopted more infection-prevention behaviors and reported significantly fewer psychological responses tied to fear and less attention paid to COVID-19-related information during summer 2020 than during CNY 2020. However, no differences were found in terms of keeping healthy, likelihood of being infected, outing risk, community risk, and physiological responses between the two time periods.

This result partially supports the first hypothesis—that more infection-prevention behaviors were adopted during T2. Participants reported heavy involvement in infection-prevention behaviors during CNY (mean = 4.16) and became even more involved during summer 2020. This finding might be related to increases in COVID cases between CNY 2020 and summer 2020 (6), along with stringent public health policy (e.g., restrictions on gatherings) concerning prevention behaviors (18, 19). Furthermore, this result is aligned with findings from the end of the SARS pandemic (52): More than 70% of the participants reported that they would wear a mask in public places and avoid going to crowded places if new SARS cases ever were to surface in HK again. People in Hong Kong might have learned from SARS in 2003 and responded quickly to COVID-19. They took infection-prevention measures during the initial stage and scored high in infection-prevention behaviors during CNY 2020. As the situation worsened, they reported taking more frequent infection-prevention measures during summer 2020.

However, participants didn't take more action to remain healthy during summer 2020 as anticipated, possibly because HK residents might have encountered barriers to increasing healthy behaviors (e.g., exercising and dieting) during the COVID-19 pandemic. For example, fitness centers were closed from mid-March 2020 to early September 2020 (19), and the living spaces in residential buildings are limited, leading to difficulties in exercising at home (53). As a result, the participants did not significantly adopt more actions to stay healthy.

The data failed to support the second research hypothesis—that people would report higher risk perception during the summer than during CNY 2020. One possible reason may be because participants maintained a high level of risk perception over time, particularly the likelihood of being infected (mean = 4.23 during T1, mean = 4.26 during T2) and community risk (mean = 4.13 during T1 and T2). The high ratings on community risk might be attributable to the severe SARS outbreak in Amoy Gardens during the SARS pandemic in 2003 (28), and the public may have perceived high risk at the beginning of the COVID-19 pandemic. Although there had been a few local COVID-19 cases in the same building before summer 2020, e.g., in Hong Mei House and Cheung Hong Estate (54), HK residents might have been alarmed throughout the COVID-19 pandemic. Future studies could investigate whether HK residents changed their perceived community risk in late 2020. During the fourth outbreak wave, starting in November 2020, many buildings reported non-epidemiologically related cases, particularly in Yau Tsim Mong District (55), which may have led to changes in people's perceptions of community risk. As such, this factor, community risk, is worthy of further investigation in future studies.

However, it is notable that high levels of risk perception can be used to explain high ratings of infection-prevention behaviors in the present study. As the health-belief model suggests, perceived susceptibility (likelihood of getting a disease) plays a role in predicting individuals' health behaviors (26). As our participants considered the high risk of COVID-19, they responded quickly and even took more actions to avoid the possibility of being infected.

The data partially supported the third research hypothesis—that participants reported reduced psychological responses to fear and paid less attention to related information during summer 2020 than during CNY. As anticipated, the public may have become desensitized after exposure to COVID-19 in the media for around half a year (44). Also, during the pandemic's initial stage, COVID-19 was new to the public, thereby triggering anxiety and fear of the unknown, and those who experienced fear viewed events with less certainty (34, 56). As accumulated studied findings became available (57), and the public might have gained more knowledge about COVID-19 during summer 2020, feelings of uncertainty and psychological responses from fear weakened. Similar findings regarding the recovery trend have been investigated in other studies in other populations. Daly and Robinson (58) compared changes in psychological distress from March to July 2020 in the U.S. and found that people's psychological distress was quite high during the initial COVID-19 outbreak in the U.S. and gradually decreased between April and summer 2020. Also, consistent with Bento et al.'s (41) findings, we found that HK people paid more attention at the beginning of the COVID-19 outbreak and reduced their attention during summer 2020. Moreover, a similar result from reduced attention to COVID-19-related information and psychological responses also may reflect a lower level of attention to relevant events (34, 59).

There were no significant differences in the physiological expressions of fear at the two time points, and on average, participants reported infrequent physiological responses to the pandemic (mean = 2.01 during CNY, mean = 1.97 during the summer period). The result might imply that the participants maintained good health and, therefore, reported fewer physiological expressions of fear.

## Conclusion

This study compared public responses to the COVID-19 pandemic during CNY 2020 to those during summer 2020. Generally, the participants reported increases in infection-prevention behaviors and decreases in their fear responses and attention paid to the pandemic during summer 2020. The participants perceived a high likelihood of being infected, outing risk, and community risk during COVID-19. Taken together, the study results suggest that the public was steered toward striking a balance between strengthening their infection-prevention behaviors and reducing their psychological responses to the pandemic during summer 2020.

However, the findings should be interpreted with caution, lest they be overgeneralized. The study didn't collect all variables that may influence people's actions, such as participants' medical history and family income. Future studies could include more demographic variables to rule out possible effects on people's actions. Second, the study was conducted in HK, so its results may not be applicable to other countries with larger outbreaks. The spread of COVID-19 in HK was successfully under control before summer 2020, but the situation was different in other countries, as indicated, for example, by De Deyn et al. (60). Third, the present study was retrospective, i.e., the participants were asked to recall their responses to the COVID-19 pandemic during CNY 2020. Even though we used a priming method to avoid potential recall bias—four pictures about CNY in the questionnaire to help trigger participants' memories of that period—future studies could conduct a longitudinal study to validate findings.

For future studies, because the COVID-19 pandemic is ongoing, monitoring public responses to it is of great importance. Moreover, further studies that investigate the underlying mechanism of people's COVID-19-related behaviors, COVID-19 risk perceptions, and attention to COVID-19-related information are necessary to explain further the changes over time.

## Data Availability Statement

The datasets for this article are not publicly available since it is an ongoing study that involves identifiable participants although no sensitive or private information were collected. Requests to access the dataset should be directed to Hui-Fang Chen (hfchen@cityu.edu.hk).

## Ethics Statement

The studies involving human participants were reviewed and approved by Human Subjects Ethics Sub-Committee, City University of Hong Kong. The patients/participants provided their written informed consent to participate in this study.

## Author Contributions

YX contributed to the design, data collection and analysis, and manuscript writing. HFC contributed to the conception, research design, data collection, and manuscript revision. WKJY contributed to data collection and analysis and manuscript revision. CWH and LC contributed to the conception, design, data collection, and manuscript revision. HYY contributed to the conception and manuscript revision. All authors contributed to the article and approved the submitted version.

## Funding

This study was partially funded by City University of Hong Kong. The grant number is 6000696.

## Conflict of Interest

The authors declare that the research was conducted in the absence of any commercial or financial relationships that could be construed as a potential conflict of interest.

## Publisher's Note

All claims expressed in this article are solely those of the authors and do not necessarily represent those of their affiliated organizations, or those of the publisher, the editors and the reviewers. Any product that may be evaluated in this article, or claim that may be made by its manufacturer, is not guaranteed or endorsed by the publisher.
