# Supplementary material for: Health-Promoting Behaviors, Risk Perceptions, and Attention to COVID-19-Related Information: Comparing People's Responses to the COVID-19 Pandemic Across Times of Chinese New Year and Summer 2020 in Hong Kong
Source: Front Public Health. 2021 Nov 23;9:688300. doi: 10.3389/fpubh.2021.688300 (PMC8650156; doi:10.3389/fpubh.2021.688300)

***Supplementary Material***

**Health-promoting behaviors**

| Infection prevention behaviors | Wore a surgical mask when going out |
| --- | --- |
|  | Washed hands more often to keep them clean |
|  | Practiced social distancing when going out |
|  | Avoided going outside unless necessary |
|  | Avoided crowds such as in shopping malls, bars, movie theaters, and hospitals |
|  | Avoided eating in restaurants |
|  | Avoided social gatherings |
|  | Avoided traveling |
| Keeping healthy | Had regular and enough sleep |
|  | Exercised regularly |
|  | Adopted a healthy diet |
|  | Avoided excessive stress and kept myself emotionally stable |

**COVID-19 risk perceptions**

| Likelihood of being infected | If I did not take protective measures, I would have been infected with COVID-19. |
| --- | --- |
|  | If my family did not take protective measures, they would have been infected with COVID-19. |
|  | If people around me did not take protective measures, they would have been infected with COVID-19. |
| Outing risk | If I took off my mask while dining in a restaurant, I would have been infected with COVID-19. |
|  | If I did not wash my hands often when going out, I would have been infected with COVID-19. |
|  | If I did not practice social distancing when going out, I would have been infected with COVID-19. |
|  | If I did not keep my home environment clean and hygienic, I would have been infected with COVID-19. |
| Community risk | If my community had the mandatory quarantine accommodations arranged by the government, my risk of being infected with COVID-19 would have increased. |
|  | If I lived in a community where there was a hospital or clinic that would treat patients with COVID-19 or would test for COVID-19, my risk of being infected with COVID-19 would have increased. |
|  | If a confirmed case of COVID-19 were found in the same building where I lived, my risk of being infected with COVID-19 would have increased. |
|  | If there were people being quarantined at home in the same building where I lived, my risk of being infected with COVID-19 would have increased. |
| Psychological responses of fear | I was most afraid of COVID-19. |
|  | It made me uncomfortable to think about COVID-19. |
|  | When watching news and stories about COVID-19 on social media, I became nervous or anxious |
|  | I was afraid of losing my life because of COVID-19. |
| Physiological expressions of fear | My hands became clammy when I thought about COVID-19. |
|  | I could not sleep because I was worrying about getting COVID-19. |
|  | My heart raced or palpitated when I thought about getting COVID-19. |
|  | When I thought I might be infected with COVID-19, my appetite became worse. |

**Priming Method:**

Four pictures about Chinese New Year


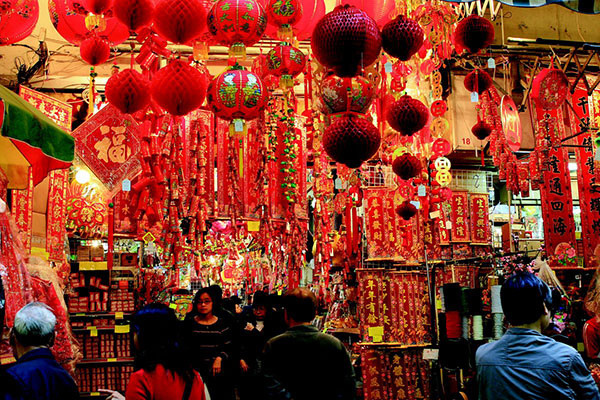
**
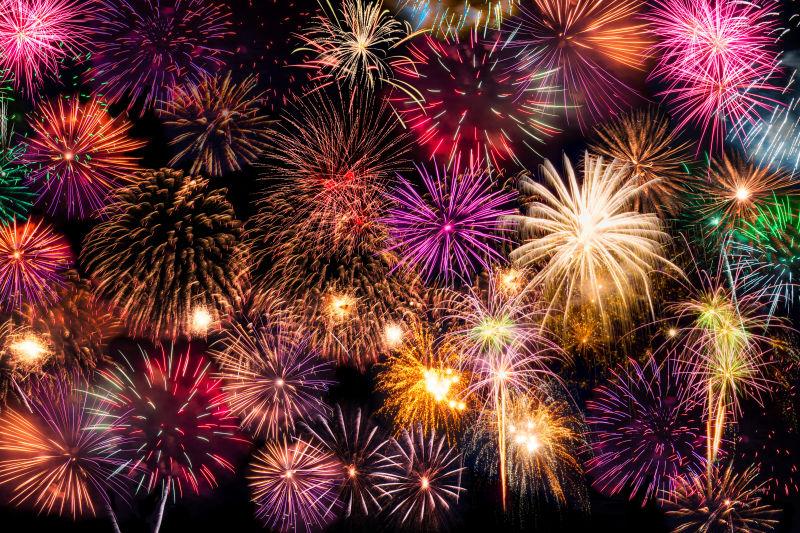
**


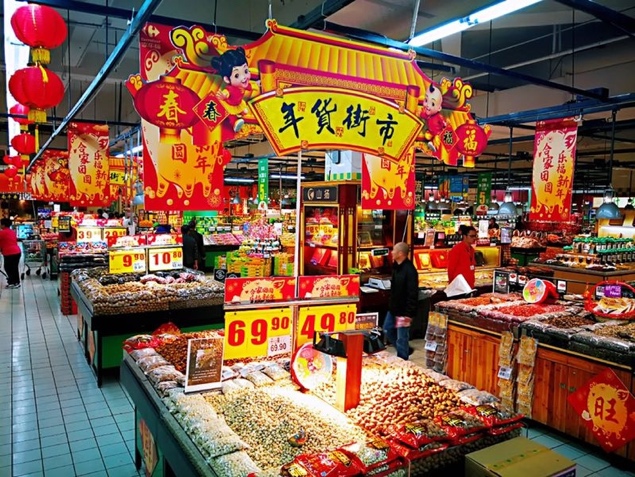

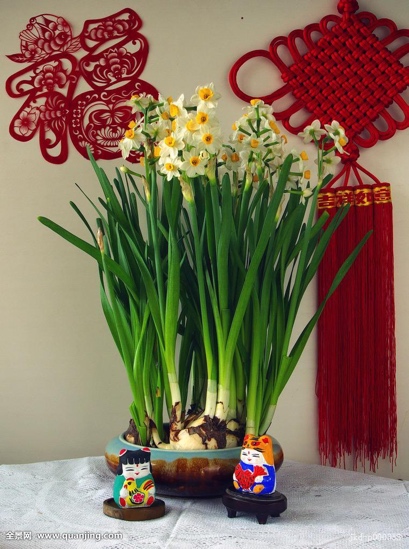

Supplement: Supplementary file 1 [file Table_1.docx]
